# Impacts of smoking on alcoholic liver disease: a nationwide cohort study

**DOI:** 10.3389/fpubh.2024.1427131

**Published:** 2024-08-07

**Authors:** Jeong-Ju Yoo, Dong Hyeon Lee, Sang Gyune Kim, Jae Young Jang, Young Seok Kim, Log Young Kim

**Affiliations:** ^1^Department of Internal Medicine, Soonchunhyang University Bucheon Hospital, Bucheon, Republic of Korea; ^2^Department of Internal Medicine, Seoul Metropolitan Government Seoul National University Boramae Medical Center, Seoul, Republic of Korea; ^3^Department of Internal Medicine, Digestive Disease Center, Institute for Digestive Research, Soonchunhyang University College of Medicine, Seoul, Republic of Korea; ^4^Department of Big Data Strategy, National Health Insurance Service, Wonju, Republic of Korea

**Keywords:** gender, smoking, alcoholic liver disease, epidemiology, cirrhosis

## Abstract

**Objectives:**

Smoking is a preventable risk factor for morbidity and mortality in patients with liver disease. This study aims to explore the additional risks of smoking in the development of alcoholic liver disease (ALD), cirrhosis, and hepatocellular carcinoma (HCC) in high-risk drinkers.

**Methods:**

Data from the National Health Insurance Service, including claims and health check-up information spanning 2011 to 2017, were used. The overall alcohol consumption was calculated, and ALD was defined based on ICD-10 codes. High-risk drinking was defined as 7 or more drinks for men and 5 or more for women, twice weekly. Half of the high-risk drinkers were smokers, decreasing in men but stable at 20% for women.

**Results:**

ALD prevalence was 0.97% in high-risk drinkers and 1.09% in high-risk drinkers who smoked, higher than 0.16% in social drinkers (*p* < 0.001). ALD incidence over 3-years was highest in high-risk drinkers who smoked (2.35%), followed by high-risk drinkers (2.03%) and social drinkers (0.35%) (*p* < 0.001). Cirrhosis and HCC followed similar patterns, with prevalence and incidence was highest in drinkers who smoked. 3-year mortality was 0.65% in high-risk drinkers who smoked, compared to 0.50% in high-risk drinkers and 0.24% in social drinkers (*p* < 0.001). Smoking increased the incidence of ALD, cirrhosis, and HCC by 1.32, 1.53, and 1.53 times, respectively (all *p* < 0.001). Gender-specific analysis revealed higher risk ratios (RR) for women in ALD, alcoholic cirrhosis, and HCC, particularly among high-risk drinkers who smoked. Women showed significantly increased RR in ALD (6.08 to 12.38) compared to men (4.18 to 4.40), and similar trends were observed for cirrhosis and HCC.

**Conclusion:**

Smoking significantly heightens the risk of ALD, cirrhosis, and HCC, especially in women, among high-risk drinkers. This emphasizes the importance of smoking cessation, particularly for female patients with ALD.

## Introduction

In the realm of liver diseases, alcoholic liver disease (ALD) has emerged as an increasingly significant health concern. ALD encompasses a spectrum of liver conditions, from fatty liver to alcoholic hepatitis and cirrhosis, predominantly associated with excessive alcohol consumption (1). The global burden of ALD is on the rise, with a worldwide prevalence of 4.8% (2). The World Health Organization (WHO) recognizes alcohol misuse as a primary risk factor for death and disability, especially among individuals aged 15 to 49 years (3). This growing significance of ALD is partly attributed to the successful development of effective antiviral treatments for chronic hepatitis B and C, which are anticipated to reduce the prevalence of these viral liver diseases and shift focus to other liver conditions like ALD (4–6).

Smoking continues to present significant global health risks, contributing to over 8 million smoking-related deaths annually. It has been identified as a substantial risk factor in the progression of various liver diseases, affecting liver health through multiple mechanisms (7, 8). For instance, smoking exacerbates oxidative stress and fibrosis in non-alcoholic fatty liver disease (NAFLD), leading to a more severe disease course (9–12). Similarly, in chronic viral hepatitis, particularly hepatitis B (HBV) and hepatitis C (HCV), smoking accelerates progression to cirrhosis and increases the risk of developing hepatocellular carcinoma (HCC) (13–15). Smoking also impacts the effectiveness of antiviral therapies and may worsen prognoses in these diseases (16, 17). Additionally, smoking has been associated with an increased risk of liver fibrosis and cirrhosis in other liver conditions, such as primary biliary cholangitis (18).

Recent studies further indicate that smoking may exacerbate liver damage in ALD. According to Marti-Aguado et al. (19), smoking is a preventable risk factor for premature morbidity and mortality, and approximately 40% of patients with liver disease have a history of smoking. The clinical evidence suggests that smoking negatively impacts the incidence and severity of fatty liver disease, fibrosis progression, HCC development, and outcomes in patients with advanced liver disease (19). The underlying mechanisms are complex and involve oxidative stress, oncogenic signals, and other pathophysiological pathways. Smoking also promotes cardiovascular disease and extrahepatic cancers in patients with liver disease, further complicating clinical outcomes.

However, the specific impact of smoking on ALD remains less clear, with current research suggesting a potential exacerbation of liver damage but lacking definitive conclusions. Previous studies have not sufficiently addressed this relationship, highlighting a significant gap in the literature. This study aims to investigate the impact of smoking on the prevalence, incidence, and prognosis of ALD using South Korean nationwide claims data. By examining this relationship, we aimed to investigate how smoking influences ALD and to inform targeted interventions and clinical management strategies.

## Materials and methods

### Data source and study population

We used the National Health Insurance Service-Health Screening Cohort (NHIS-HEALs) database in South Korea, which operates a universal health coverage system with mandatory social health insurance, covering approximately 98% of the total population. This study is a prospective cohort study, tracking groups with different health habits over time to observe outcomes. Due to security and data capacity concerns, the data for the entire NHIS-HEALs population was not provided. Instead, we selected a sample cohort representing 10% of the NHIS population randomly each year from 2011 to 2017 (Supplementary Table S1). This sample cohort was designed to mirror the age and gender structure of the entire NHIS dataset, making it representative of the entire Korean population. The sample size each year ranged from 3,769,258 individuals in 2011 to 3,982,051 individuals in 2017. The follow-up period varies: Table 1 represents a 10-year follow-up cohort, while the other tables and graphs represent data from a 3-year follow-up period.

**Table 1:** Risk of alcoholic liver disease, cirrhosis, and hepatocellular carcinoma.

Factor	Alcoholic liver disease		Liver cirrhosis		Hepatocellular carcinoma	
	HR (95% CI)	*p*	HR (95% CI)	*p*	HR (95% CI)	*p*
High risk drinking	3.478 (3.373–3.587)	<0.001	1.841 (1.743–1.945)	<0.001	1.375 (1.263–1.497)	<0.001
Smoking	1.325 (1.293–1.357)	<0.001	1.534 (1.457–1.615)	<0.001	1.532 (1.411–1.664)	<0.001
Physical activity	0.912 (0.891–0.933)	<0.001	0.821 (0.782–0.862)	<0.001	0.857 (0.792–0.927)	<0.001
Older than 40 years	2.178 (2.126–2.232)	<0.001	5.093 (4.778–5.429)	<0.001	6.341 (5.648–7.118)	<0.001
Male	2.343 (2.250–2.439)	<0.001	1.763 (1.636–1.900)	<0.001	2.460 (2.152–2.812)	<0.001
Body mass index (kg/m^2^)	1.020 (1.017–1.024)	<0.001	0.974 (0.966–0.982)	<0.001	1.000 (0.987–1.013)	0.994
Diabetes mellitus	1.323 (1.244–1.407)	<0.001	1.624 (1.465–1.800)	<0.001	1.629 (1.396–1.900)	<0.001
Hepatitis B virus infection	1.433 (1.353–1.518)	<0.001	9.933 (9.384–10.514)	<0.001	21.149 (19.524–22.908)	<0.001
Hepatitis C virus infection	2.208 (2.024–2.409)	<0.001	2.149 (1.893–2.440)	<0.001	1.756 (1.458–2.116)	<0.001

The database comprises de-identified information, including demographic and claimed data based on the International Classification of Diseases, 10th revision (ICD-10). The NHIS medical check-up database includes baseline information such as demographic data, lifestyle and behavior data (e.g., smoking, drinking habits), physical examination results, laboratory test results, and medical history. Over time, it records subsequent health examinations, medical treatments, and disease incidence. The Institutional Review Board approved the study (IRB No, SCHBC 2023–05-007, approval date 23-May-2023). Informed consent was waived by the IRB since only de-identified information was used. Our study adhered to the ethical guidelines outlined in the World Medical Association Declaration of Helsinki.

#### Inclusion and exclusion criteria

For this study, we included adults aged 20 years and older who had undergone health screenings conducted by the NHIS, which provided comprehensive data on alcohol consumption and smoking habits. For the analysis related to ALD incidence and prevalence, we included individuals aged 20 years and older who had a diagnosis of ALD based on ICD-10 codes K70 (K700, K701, K702, K703, K704, and K709). We excluded individuals with a diagnosis of HCC or liver cirrhosis prior to the study period and those with incomplete data or missing key variables required for the analysis. The study did not impose restrictions based on age or residential status. In South Korea, the NHIS provides coverage for nearly the entire population, with private insurance representing a very small fraction. As a result, our study did not separately account for those with private insurance, focusing exclusively on data obtained from the NHIS.

### Definitions of alcohol drinking, smoking, and physical activity

High-risk drinking was defined as consuming alcohol more than twice a week, with men exceeding 7 standard drinks and women surpassing 5 standard drinks, according to the guidelines of South Korea’s Ministry of Health and Welfare (20). One standard drink is equivalent to 7 grams of pure alcohol, as per the South Korean alcohol consumption guideline (21). Social drinkers served as the control group for high-risk drinking, defined as individuals who drank once a week, with men consuming 6 or fewer standard drinks and women consuming 4 or fewer standard drinks (20). A smoker was defined as an individual who has smoked a total of 5 packs (100 cigarettes) or more in their lifetime and currently engages in smoking (22). Physical activity was characterized by participating in high-intensity exercises (running, aerobics, fast-speed cycling, hiking, etc.) for more than 20 min or engaging in moderate activities (brisk walking, tennis, moderate-speed cycling, etc.) for more than 30 min at least once a week (23).

### Outcomes and covariates

The study’s primary outcomes were the prevalence and incidence of ALD, alcoholic cirrhosis, and HCC. ALD was defined as having a history of outpatient treatment more than twice or being hospitalized more than once, with the main diagnosis code falling within ICD-10 codes K70 (K700, K701, K702, K703, K704, and K709). Liver cirrhosis was defined by ICD-10 codes K74, K702, and K703, while HCC was defined by C220. Mortality was considered in cases where death was reported, regardless of the cause. Incidence was calculated as the occurrence of a new outcome during the 3-year follow-up of the sample cohort. To assess the impact of smoking on the incidence of liver disease, adjustments were made for alcohol consumption, physical activity, age, gender, obesity evaluated by body mass index (BMI), diabetes, hepatitis B virus (HBV) infection, and hepatitis C virus (HCV) infection. BMI was assessed as a continuous variable, diabetes mellitus (DM) was defined by ICD-10 codes E08 to E13, HBV infection was defined by ICD-10 codes B180 and B181, and HCV infection was defined by ICD-10 code B182.

### Statistical analysis

Continuous variables were reported as means ± standard deviations (SDs), while categorical variables were expressed as proportions unless otherwise specified. Group differences were analyzed using Student’s *t*-test for continuous variables and the *χ*^2^ test for categorical variables. The impact of smoking on each group was assessed through relative risk (RR). Multivariate analysis using Cox regression test was used to determine the effect of smoking on each outcome. Statistical analyses were performed using SAS version 9.4 (SAS Institute, Cary, NC, United States) and R version 3.2.3 (The R Foundation for Statistical Computing, Vienna, Austria, http://www.Rproject.org). A two-sided *p*-value <0.05 was considered statistically significant.

## Results

### Alcohol consumption rate

The proportion of social drinkers in the sample cohort was 15.4% in 2011, gradually increasing to 16.4% in 2017 (Supplementary Figure S1) (*p* for trend <0.001). The proportion was consistently higher among men than women (all *p* < 0.001) and exhibited an upward trend for both genders annually (*p* for trend <0.001). Conversely, high-risk drinkers constituted approximately 14.7% of the sample cohort, maintaining a steady rate from 2011 to 2017. The prevalence of high-risk drinking was higher in men than women, with the rate decreasing annually for men and increasing for women.

### Smoking rate according to amount of alcohol consumption

The proportion of smokers among social drinkers was 22.82% in 2011, progressively declining each year to 17.79% in 2017 (Supplementary Figure S2) (*p* for trend <0.001). The smoking prevalence was notably higher in men than in women (all *p* < 0.001) but exhibited a decreasing trend for both genders (*p* for trend <0.001). Concurrently, the smoking rate among high-risk drinkers significantly increased compared to social drinkers (*p* < 0.001), with an average of 50% of high-risk drinkers being smokers. While the smoking rate among men was higher than that of women, it decreased annually for men, whereas it remained constant at an average of 20% among women.

### Smoking and alcoholic liver disease

We investigated the impact of smoking on the prevalence and incidence of ALD. The prevalence of ALD increased across social drinkers (0.16%) and high-risk drinkers (0.97%), reaching the highest value in the high-risk drinker with smoking group (1.09%) (Supplementary Table S2) (*p* < 0.001). Across all three groups, the prevalence of ALD was consistently higher in men than in women (men: 0.26, 1.12, 1.12%; women: 0.05, 0.37, 0.76%) (*p* < 0.001). The incidence of ALD over a 3-year period was 0.35% in the social drinker group, rising to 2.03% in the high-risk drinker group, and further to 2.35% in the high-risk drinker with smoking group (Supplementary Table S6) (*p* < 0.001). While the overall incidence of ALD was higher in men than in women, the risk increased significantly in women when transitioning from the high-risk drinker group to the high-risk drinker with smoking group (men: 2.33 to 2.41%; women: 0.82 to 1.67%).

### Smoking and alcoholic liver cirrhosis

Subsequently, we investigated the impact of smoking on the prevalence and incidence of alcoholic cirrhosis. The prevalence of cirrhosis in the social drinker, high-risk drinker, and high-risk drinker with smoking groups was 0.10, 0.19, and 0.22%, respectively (Supplementary Table S3) (*p* < 0.001). We then explored the incidence of alcoholic cirrhosis over a 3-year period in the sample cohort. At the 3-year follow-up, the highest incidence rate of ALD was observed in the high-risk drinker with smoking group at 0.52%, followed by the high-risk drinker group at 0.42%, and the social drinker group at 0.18% (Supplementary Table S7) (*p* < 0.001).

### Smoking and hepatocellular carcinoma

There was a noteworthy disparity in the prevalence of HCC among the 3 groups (Supplementary Table S4). It was at its lowest in the social drinker group (0.033%), with a significant increase in both the high-risk drinker (0.042%) and high-risk drinker with smoking groups (0.045%) (*p* < 0.001). Similarly, the incidence of HCC exhibited a significant increase in the high-risk drinker with smoking group (0.14%), followed by the high-risk drinker group (0.12%) and the social drinker group (0.03%) (Supplementary Table S8) (*p* < 0.001). The incidence of HCC was consistently higher in men than in women across all groups (all *p* < 0.001).

### Smoking and 3-year mortality

The 3-year mortality rate in the social drinker group was 0.24%, escalating to 0.50 and 0.65% in the high-risk drinker and high-risk drinker with smoking groups, respectively (Supplementary Table S5) (*p* < 0.001). Across all groups, men exhibited a higher 3-year mortality rate than women (men: 0.36, 0.57, 0.67%; women: 0.11, 0.22, 0.42%) (all *p* < 0.001).

### Multivariate analysis of the effects of smoking on the development of ALD, cirrhosis, and HCC

We conducted Cox regression multivariate analysis to assess the impact of smoking on the occurrence of ALD, alcoholic cirrhosis, and HCC (Table 1). After adjusting for alcohol consumption, physical activity, gender, old age, obesity by body mass index, HBV infection, and HCV infection, smoking increased the incidence of ALD, cirrhosis, and HCC by 1.32 (Hazard Ration; HR 1.325, 95% CI 1.293–1.357, *p* < 0.001), 1.53 (HR 1.534, 95% CI 1.457–1.615, p < 0.001), and 1.53 times (HR 1.532, 95% CI 1.411–1.664, *p* < 0.001), respectively.

### Smoking and female vulnerability

**Figure 1:**
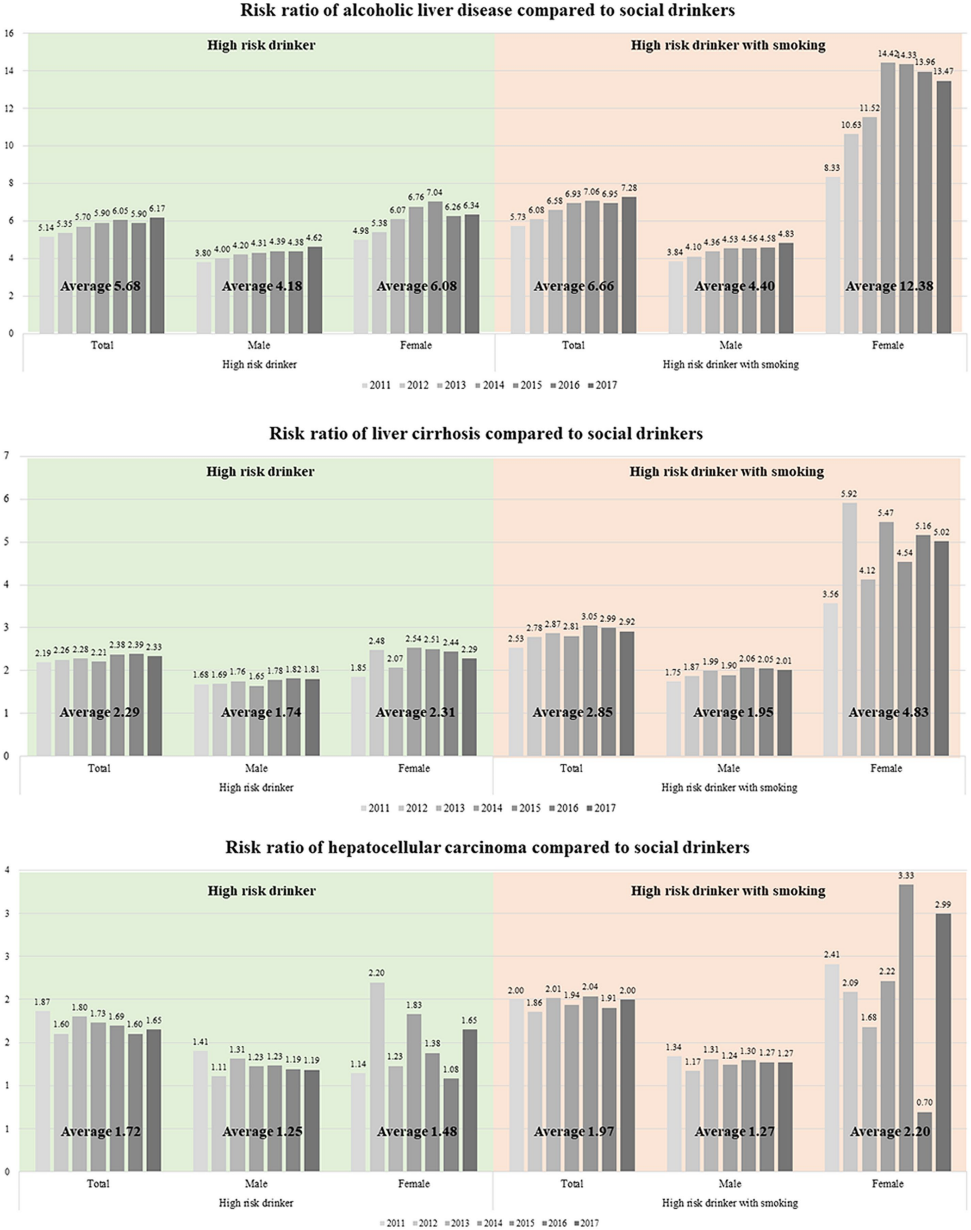
Risk ratio of **(A)** alcoholic liver disease, **(B)** liver cirrhosis, and **(C)** hepatocellular carcinoma compared with social drinkers.

Lastly, stratified by gender, we computed the risk ratio for developing ALD, alcoholic cirrhosis, and HCC. Smoking risks exhibited gender-based variations (Figure 1). In terms of ALD incidence, the RR value in the high-risk drinker group was higher in women than in men (6.08 vs. 4.18). This risk further escalated in the high-risk drinker with smoking group. Notably, for women, the RR significantly increased from 6.08 to 12.38, compared to a change from 4.18 to 4.40 for men (Figure 1A). Women were also more susceptible to smoking than men in developing alcoholic cirrhosis (Figure 1B) or HCC (Figure 1C). In the high-risk drinker with smoking group, the RR for women developing cirrhosis increased from 2.31 to 4.83, representing a larger increase than the change in men’s RR from 1.74 to 1.95.

## Discussion

As our understanding of liver disease advances, especially with the decreasing prevalence of viral hepatitis due to medical advancements, the emphasis on ALD becomes more apparent. This underscores the need to explore modifiable factors that may worsen ALD, with smoking as a key concern. Unlike other liver diseases where the harmful effects of smoking are recognized, the mechanisms and outcomes of smoking in the context of ALD remain unclear. Our study affirms that smoking elevates the prevalence and incidence of ALD, along with the occurrence of HCC, with a notably significant risk increase in females.

It is well-recognized that smoking adversely affects various liver diseases, warranting clinical attention due to its status as a representative modifiable factor. The impact of smoking on liver disease, categorized by etiology, is elucidated as follows: In HBV patients, heavy smoking (> − 20 cigarettes/day) emerges as a significant risk factor exacerbating the progression to liver cirrhosis (24). Also, smoking has been reported to have a negative effect on fibrosis regression in HBV patients undergoing antiviral therapy (25). Moreover, smoking was associated with a high viral load and severe liver inflammation in HBV patients (15). Smoking is associated with an increased inflammatory activity grade, and smoking cessation has been shown to contribute to improved fibrosis after HCV clearance (26). The causal relationship between NAFLD and smoking has been firmly established in numerous cross-sectional and cohort studies. Smoking exacerbates steatosis and fibrosis progression in NAFLD patients and contributes to increased cardiovascular disease (CVD) mortality in this population (27, 28). Furthermore, it heightens the risk of progression to liver cirrhosis in MAFLD patients (12). Lastly, concerning HCC, smoking is linked to an increased incidence, recurrence, and mortality from HCC (29–32).

In this study, we used social drinker group as the control group for high-risk drinking group. Non-drinkers were not considered as the control group due to their relatively small proportion in the study sample and the high deviation in their baseline characteristics. This group includes individuals who may abstain from alcohol due to chronic illnesses or frailty. Instead, social drinkers, defined as individuals who drank once a week, with men consuming 6 or fewer standard drinks and women consuming 4 or fewer standard drinks, were chosen as the control group. Social drinkers are more likely to lead healthier lifestyles and can voluntarily control their alcohol consumption, making them a more appropriate comparison group for high-risk drinkers.

The first finding of our study was to analyze the effect of smoking on ALD in a nationwide cohort. Although the association between various liver diseases and smoking has been well-documented, the impact of smoking on ALD has been relatively less explored. In 1994, smoking was reported as a faint risk factor for cirrhosis in 115 alcoholic liver cirrhosis patients and 167 control groups. In 2018, smoking was associated with increased mortality in cirrhosis patients with heavy alcohol consumption (33, 34). Our study found that smoking can increase the prevalence, incidence, and mortality of ALD. Particularly in heavy-drinker patients, smoking elevated the incidence of ALD by 1.3 times, liver cirrhosis by 1.5 times, and HCC by 1.4 times. To date, our study stands as the only large-scale investigation demonstrating a robust association between smoking and ALD.

The hypothesis that smoking exacerbates ALD involves several interconnected physiological mechanisms. Firstly, both alcohol and tobacco smoke introduce a range of toxins to the liver, including acetaldehyde from alcohol metabolism and harmful chemicals from tobacco (35). This dual exposure significantly amplifies the liver’s toxic burden, resulting in more severe damage. Secondly, the combined effect of smoking and alcohol consumption heightens oxidative stress and inflammation in the liver, further aggravating liver cell damage (36). Additionally, smoking impairs the liver’s ability to regenerate, hindering its healing process and exacerbating the damage caused by alcohol (37). Furthermore, smoking accelerates the progression of liver fibrosis and scarring, common consequences of chronic alcohol consumption, leading to a faster progression of liver diseases such as cirrhosis (12). Lastly, both smoking and alcohol compromise the immune system, diminishing the body’s ability to combat infections and repair liver damage (38).

The second finding of our study is that the effects of smoking differ by gender, with women being more vulnerable. Although the number of patients with ALD, HCC, and death was higher in men, the calculated RR for women increased more markedly than that for men. Considering that men are a common risk factor for most liver diseases, the discovery that smoking, which increases the risk in women, is a unique finding. The heightened vulnerability of women to the harmful effects of smoking on liver health, particularly in the context of ALD, can be linked to several key factors (39). Women’s livers may metabolize the toxins in tobacco smoke differently than men, potentially leading to greater harm, due to variations in enzyme activity. Hormonal differences, particularly involving estrogen, might exacerbate the impact of smoking-related toxins on the liver (40). Additionally, women might exhibit a heightened inflammatory response to smoking, accelerating liver damage (41). These factors, combined with the already harmful effects of alcohol on the liver, result in a more pronounced risk for women in developing ALD when they smoke, highlighting gender-specific susceptibility. Therefore, clinicians must check the smoking status of female patients with ALD and thoroughly educate them about smoking cessation if they smoke, which is beneficial for long-term prognosis. While our study highlights the higher relative risks observed in women, it is essential to address the significantly higher proportions of high-risk drinking and smoking among men. National policies should therefore be designed to target both men and women to effectively reduce the overall risk of liver diseases. By incorporating gender-specific strategies, public health initiatives can more accurately address the vulnerabilities and behaviors of each group, ultimately leading to a more significant impact on liver disease prevention and health promotion.

Our study has several limitations. First, there is potential selection bias arises from the random annual selection of only 5% of the total population, which may not fully represent the entire population’s drinking and smoking behaviors. Additionally, the observational nature of the study restricts the ability to establish causation between alcohol consumption, smoking, and liver disease outcomes. Reliance on self-reported data for alcohol consumption and smoking habits introduces the possibility of reporting bias, such as underreporting or over reporting. Furthermore, the study’s findings are limited by the absence of detailed information on other potential confounding factors, such as dietary habits, genetic predispositions, and socioeconomic status that could influence the development of ALD and HCC. Our study utilized a 10% subsample of the NHIS-HEALs cohort, specifically designed to reflect the age and gender structure of the entire Korean population. While additional socio-demographic factors such as education, household income, and marital status were not directly stratified, the large sample size and random stratified sampling method employed ensures that these factors are indirectly represented. It is acknowledged that these factors can influence health behaviors and outcomes, and future studies should aim to include these variables more explicitly. However, the current methodology provides a robust reflection of the population characteristics, ensuring the reliability and generalizability of the findings.

In conclusion, smoking is a modifiable factor that significantly increases ALD, cirrhosis, HCC, and death in the high-risk drinkers. Given the heightened risk in women, smoking cessation is particularly essential in female ALD patients. Our results need to be confirmed through future prospective studies, and additional research is needed to determine whether smoking cessation can improve this prognosis. Lastly, experimental research is needed to examine why women are more vulnerable to ALD when they smoke compared to men.

## Data availability statement

The raw data supporting the conclusions of this article will be made available by the authors, without undue reservation.

## Ethics statement

The studies involving humans were approved by the Institutional Review Board (IRB No, SCHBC 2023–05-007, approval date 23-May-2023). Our study adhered to the ethical guidelines outlined in the World Medical Association Declaration of Helsinki. The studies were conducted in accordance with the local legislation and institutional requirements. The ethics committee/institutional review board waived the requirement of written informed consent for participation from the participants or the participants’ legal guardians/next of kin because informed consent was waived by the IRB since only de-identified information was used.

## Author contributions

J-JY: Formal analysis, Writing – original draft. DL: Investigation, Writing – original draft. SK: Investigation, Writing – original draft. JJ: Investigation, Writing – original draft. YK: Investigation, Writing – original draft. LK: Conceptualization, Writing – review & editing.

## References

[1] Dunn W Shah VH. Pathogenesis of alcoholic liver disease. Clin Liver Dis. (2016) 20:445–56. doi: 10.1016/j.cld.2016.02.004 27373608 PMC4933837

[2] Rehm J Samokhvalov AV Shield KD. Global burden of alcoholic liver diseases. J Hepatol. (2013) 59:160–8. doi: 10.1016/j.jhep.2013.03.007 23511777

[3] World Health Organization. Global status report on alcohol and health 2018. Geneva: World Health Organization (2018).

[4] Nguyen LBL Lemoine M Ndow G Ward ZJ Hallet TB D'Alessandro U. Treat all versus targeted strategies to select HBV-infected people for antiviral therapy in the Gambia, West Africa: a cost-effectiveness analysis. Lancet Glob Health. (2024) 12:e66–78. doi: 10.1016/S2214-109X(23)00467-9, PMID: 38097300

[5] Muzica CM Stanciu C Huiban L Singeap AM Sfarti C Zenovia S. Hepatocellular carcinoma after direct-acting antiviral hepatitis C virus therapy: a debate near the end. World J Gastroenterol. (2020) 26:6770–81. doi: 10.3748/wjg.v26.i43.6770, PMID: 33268960 PMC7684455

[6] Ahn YH Lee H Han JE Cho HJ Cheong JY Park B. Effect of direct-acting antivirals for hepatitis C virus-related hepatocellular carcinoma recurrence and death after curative treatment. J Liver Cancer. (2022) 22:125–35. doi: 10.17998/jlc.2022.05.24, PMID: 37383412 PMC10035739

[7] Rutledge SM Asgharpour A. Smoking and liver disease. Gastroenterol Hepatol. (2020) 16:617–25. PMID: 34035697 PMC8132692

[8] Hezode C Lonjon I Roudot-Thoraval F Mavier JP Pawlotsky JM Zafrani ES. Impact of smoking on histological liver lesions in chronic hepatitis C. Gut. (2003) 52:126–9. doi: 10.1136/gut.52.1.126, PMID: 12477773 PMC1773517

[9] Chen QF Zhou XD Fang DH Sun YJ Zhao Q Huang JH. Impact of non-alcoholic fatty liver disease and smoking on colorectal polyps. Oncotarget. (2017) 8:74927–35. doi: 10.18632/oncotarget.20462, PMID: 29088835 PMC5650390

[10] Zein CO Unalp A Colvin R Liu YC AJ MC Nonalcoholic Steatohepatitis Clinical Research N. Smoking and severity of hepatic fibrosis in nonalcoholic fatty liver disease. J Hepatol. (2011) 54:753–9. doi: 10.1016/j.jhep.2010.07.040. PMID 21126792, PMID: 21126792 PMC3060962

[11] Mumtaz H Hameed M Sangah AB Zubair A Hasan M. Association between smoking and non-alcoholic fatty liver disease in Southeast Asia. Front Public Health. (2022) 10:1008878. doi: 10.3389/fpubh.2022.1008878, PMID: 36582387 PMC9793992

[12] Yoo JJ Park MY Cho EJ Yu SJ Kim SG Kim YJ. Smoking increases the risk of hepatocellular carcinoma and cardiovascular disease in patients with metabolic-associated fatty liver disease. J Clin Med. (2023) 12:336. doi: 10.3390/jcm12093336, PMID 37176776 37176776 PMC10179445

[13] Zhang XF Wei T Liu XM Liu C Lv Y. Impact of cigarette smoking on outcome of hepatocellular carcinoma after surgery in patients with hepatitis B. PLoS One. (2014) 9:e85077. doi: 10.1371/journal.pone.0085077, PMID: 24454795 PMC3893178

[14] Chuang SC Lee YC Hashibe M Dai M Zheng T Boffetta P. Interaction between cigarette smoking and hepatitis B and C virus infection on the risk of liver cancer: a meta-analysis. Cancer Epidemiol Biomarkers Prev. (2010) 19:1261–8. doi: 10.1158/1055-9965.EPI-09-1297, PMID: 20447919 PMC4170071

[15] Wang YH Chuang YH Wu CF Jan MC Wu WJ Lin CL. Smoking and hepatitis B virus-related hepatocellular carcinoma risk: the mediating roles of viral load and alanine aminotransferase. Hepatology. (2019) 69:1412–25. doi: 10.1002/hep.30339, PMID: 30382583

[16] Qiu F Liang CL Liu H Zeng YQ Hou S Huang S. Impacts of cigarette smoking on immune responsiveness: up and down or upside down? Oncotarget. (2017) 8:268–84. doi: 10.18632/oncotarget.13613, PMID: 27902485 PMC5352117

[17] Pessione F Ramond MJ Njapoum C Duchatelle V Degott C Erlinger S. Cigarette smoking and hepatic lesions in patients with chronic hepatitis C. Hepatology. (2001) 34:121–5. doi: 10.1053/jhep.2001.25385, PMID: 11431742

[18] Wijarnpreecha K Werlang M Panjawatanan P Pungpapong S Lukens FJ Harnois DM. Smoking & risk of advanced liver fibrosis among patients with primary biliary cholangitis: a systematic review & meta-analysis. Indian J Med Res. (2021) 154:806–12. doi: 10.4103/ijmr.IJMR_639_19, PMID: 35662085 PMC9347256

[19] Marti-Aguado D Clemente-Sanchez A Bataller R. Cigarette smoking and liver diseases. J Hepatol. (2022) 77:191–205. doi: 10.1016/j.jhep.2022.01.016 35131406

[20] Harris E. FDA approves RSV monoclonal antibody for infants and young children. JAMA. (2023) 330:586. doi: 10.1001/jama.2023.13137 37494062

[21] Lee S Kim JS Jung JG Oh MK Chung TH Kim J. Korean alcohol guidelines for moderate drinking based on facial Flushing. Korean J Fam Med. (2019) 40:204–11. doi: 10.4082/kjfm.19.0059, PMID: 31302995 PMC6669389

[22] Ryan H Trosclair A Gfroerer J. Adult current smoking: differences in definitions and prevalence estimates--NHIS and NSDUH, 2008. J Environ Public Health. (2012) 2012:918368:1–11. doi: 10.1155/2012/918368 22649464 PMC3357540

[23] Seo YB Oh YH Yang YJ. Current status of physical activity in South Korea. Korean J Fam Med. (2022) 43:209–19. doi: 10.4082/kjfm.22.0099, PMID: 35903044 PMC9334717

[24] Mori M Hara M Wada I Hara T Yamamoto K Honda M. Prospective study of hepatitis B and C viral infections, cigarette smoking, alcohol consumption, and other factors associated with hepatocellular carcinoma risk in Japan. Am J Epidemiol. (2000) 151:131–9. doi: 10.1093/oxfordjournals.aje.a010180, PMID: 10645815

[25] Xiong M Li J Yang S Zeng F Ji Y Liu J. Impacts of cigarette smoking on liver fibrosis and its regression under therapy in male patients with chronic hepatitis B. Liver Int. (2019) 39:1428–36. doi: 10.1111/liv.14108 30920714

[26] Balmaceda JB Aepfelbacher J Belliveau O Chaudhury CS Chairez C McLaughlin M. Long-term changes in hepatic fibrosis following hepatitis C viral clearance in patients with and without HIV. Antivir Ther. (2019) 24:451–7. doi: 10.3851/IMP3327, PMID: 31359874 PMC8296308

[27] Akhavan Rezayat A Dadgar Moghadam M Ghasemi Nour M Shirazinia M Ghodsi H Rouhbakhsh Zahmatkesh MR. Association between smoking and non-alcoholic fatty liver disease: a systematic review and meta-analysis. SAGE Open Med. (2018) 6:205031211774522. doi: 10.1177/2050312117745223 PMC5788091 29399359

[28] Charatcharoenwitthaya P Karaketklang K Aekplakorn W. Cigarette smoking increased risk of overall mortality in patients with non-alcoholic fatty liver disease: a Nationwide population-based cohort study. Front Med. (2020) 7:604919. doi: 10.3389/fmed.2020.604919, PMID: 33365321 PMC7750535

[29] Petrick JL Campbell PT Koshiol J Thistle JE Andreotti G Beane-Freeman LE. Tobacco, alcohol use and risk of hepatocellular carcinoma and intrahepatic cholangiocarcinoma: the liver Cancer pooling project. Br J Cancer. (2018) 118:1005–12. doi: 10.1038/s41416-018-0007-z, PMID: 29520041 PMC5931109

[30] Abdel-Rahman O Helbling D Schob O Eltobgy M Mohamed H Schmidt J. Cigarette smoking as a risk factor for the development of and mortality from hepatocellular carcinoma: an updated systematic review of 81 epidemiological studies. J Evid Based Med. (2017) 10:245–54. doi: 10.1111/jebm.12270 28891275

[31] Kim DY. Changing etiology and epidemiology of hepatocellular carcinoma: Asia and worldwide. J Liver Cancer. (2024) 24:62–70. doi: 10.17998/jlc.2024.03.13, PMID: 38523466 PMC10990659

[32] Lee J Choi JY Lee SK. Heavy smoking increases early mortality risk in patients with hepatocellular carcinoma after curative treatment. J Liver Cancer. (2024). doi: 10.17998/jlc.2024.06.02, PMID: 38852989 PMC11449571

[33] Sezgin Vatansever ZP Deniz S Unsal B. Effect of smoking on cirrhosis with heavy alcohol consumption. Scholars J Appl Med Sci. (2018) 6:3596–601.

[34] Corrao G Lepore AR Torchio P Valenti M Galatola G D'Amicis A. The effect of drinking coffee and smoking cigarettes on the risk of cirrhosis associated with alcohol consumption. A case-control study. Provincial Group for the Study of chronic liver disease. Eur J Epidemiol. (1994) 10:657–64. doi: 10.1007/BF01719277, PMID: 7672043

[35] Seitz HK Becker P. Alcohol metabolism and cancer risk. Alcohol Res Health. (2007) 30:38–41. PMID: 17718399 PMC3860434

[36] Bailey SM Mantena SK Millender-Swain T Cakir Y Jhala NC Chhieng D. Ethanol and tobacco smoke increase hepatic steatosis and hypoxia in the hypercholesterolemic apoE(−/−) mouse: implications for a "multihit" hypothesis of fatty liver disease. Free Radic Biol Med. (2009) 46:928–38. doi: 10.1016/j.freeradbiomed.2009.01.003, PMID: 19280709 PMC2775483

[37] Lv Y So KF Xiao J. Liver regeneration and alcoholic liver disease. Ann Transl Med. (2020) 8:567. doi: 10.21037/atm.2020.02.168, PMID: 32775368 PMC7347779

[38] Barr T Helms C Grant K Messaoudi I. Opposing effects of alcohol on the immune system. Prog Neuro Psychopharmacol Biol Psychiatry. (2016) 65:242–51. doi: 10.1016/j.pnpbp.2015.09.001, PMID: 26375241 PMC4911891

[39] Bizzaro D Becchetti C Trapani S Lavezzo B Zanetto A D'Arcangelo F. Influence of sex in alcohol-related liver disease: pre-clinical and clinical settings. United European. Gastroenterol J. (2023) 11:218–27. doi: 10.1002/ueg2.12370. PMID 36866682, PMID: 36866682 PMC10039798

[40] Mueck AO Seeger H. Smoking, estradiol metabolism and hormone replacement therapy. Curr Med Chem Cardiovasc Hematol Agents. (2005) 3:45–54. doi: 10.2174/1568016052773270 15638743

[41] Kim JW Zhou Z Yun H Park S Choi SJ Lee SH. Cigarette smoking differentially regulates inflammatory responses in a mouse model of nonalcoholic steatohepatitis depending on exposure time point. Food Chem Toxicol. (2020) 135:110930. doi: 10.1016/j.fct.2019.110930, PMID: 31678261

