# Supplementary material for: Impacts of smoking on alcoholic liver disease: a nationwide cohort study
Source: Front Public Health. 2024 Aug 7;12:1427131. doi: 10.3389/fpubh.2024.1427131 (PMC11335641; doi:10.3389/fpubh.2024.1427131)
Supplement: Supplementary file 1 [file Table_1.docx]

**Supplementary** **Table 1.** National Health Insurance Service-Health Screening Cohort (NHIS-HEALs) and 10% sample cohort

|  |  | 2011 | | 2012 | | 2013 | | 2014 | | 2015 | | 2016 | | 2017 | | |
| --- | --- | --- | --- | --- | --- | --- | --- | --- | --- | --- | --- | --- | --- | --- | --- | --- |
| Sex | Age | Population | Sample (10%) | Population | Sample (10%) | Population | Sample (10%) | Population | Sample (10%) | Population | Sample (10%) | Population | Sample (10%) | Population | Sample (10%) |  |
| Male | 20 - 29 | 3,513,630 | 351,363 | 3,462,092 | 346,209 | 3,438,604 | 343,860 | 3,455,018 | 345,502 | 3,492,794 | 349,279 | 3,526,733 | 352,673 | 3,556,649 | 355,665 |  |
|  | 30 - 39 | 4,193,211 | 419,321 | 4,143,117 | 414,312 | 4,085,613 | 408,561 | 4,000,925 | 400,093 | 3,917,274 | 391,727 | 3,850,487 | 385,049 | 3,778,265 | 377,827 |  |
|  | 40 - 49 | 4,435,055 | 443,506 | 4,437,927 | 443,793 | 4,452,694 | 445,269 | 4,474,485 | 447,449 | 4,459,325 | 445,933 | 4,418,590 | 441,859 | 4,376,817 | 437,682 |  |
|  | 50 - 59 | 3,592,137 | 359,214 | 3,776,653 | 377,665 | 3,909,235 | 390,924 | 4,020,937 | 402,094 | 4,097,255 | 409,726 | 4,155,589 | 415,559 | 4,198,174 | 419,817 |  |
|  | 60 - 69 | 1,983,828 | 198,383 | 2,025,529 | 202,553 | 2,094,829 | 209,483 | 2,194,269 | 219,427 | 2,342,576 | 234,258 | 2,502,779 | 250,278 | 2,648,477 | 264,848 |  |
|  | 70 - 79 | 1,096,507 | 109,651 | 1,183,270 | 118,327 | 1,258,609 | 125,861 | 1,305,432 | 130,543 | 1,337,821 | 133,782 | 1,369,560 | 136,956 | 1,426,917 | 142,692 |  |
|  | Sum | 18,814,368 | 1,881,438 | 19,028,588 | 1,902,859 | 19,239,584 | 1,923,958 | 19,451,066 | 1,945,108 | 19,647,045 | 1,964,705 | 19,823,738 | 1,982,374 | 19,985,299 | 1,998,531 |  |
| Female | 20 - 29 | 3,246,006 | 324,601 | 3,180,980 | 318,098 | 3,137,257 | 313,726 | 3,130,169 | 313,017 | 3,146,887 | 314,689 | 3,169,220 | 316,922 | 3,194,583 | 319,458 |  |
|  | 30 - 39 | 4,032,981 | 403,298 | 3,990,002 | 399,000 | 3,933,220 | 393,322 | 3,846,194 | 384,619 | 3,760,633 | 376,063 | 3,688,221 | 368,822 | 3,610,380 | 361,038 |  |
|  | 40 - 49 | 4,273,990 | 427,399 | 4,275,492 | 427,549 | 4,303,860 | 430,386 | 4,336,399 | 433,640 | 4,323,989 | 432,399 | 4,297,638 | 429,764 | 4,263,677 | 426,368 |  |
|  | 50 - 59 | 3,583,577 | 358,358 | 3,760,656 | 376,066 | 3,877,090 | 387,709 | 3,979,647 | 397,965 | 4,051,530 | 405,153 | 4,092,336 | 409,234 | 4,131,629 | 413,163 |  |
|  | 60 - 69 | 2,166,711 | 216,671 | 2,192,207 | 219,221 | 2,248,556 | 224,856 | 2,335,678 | 233,568 | 2,482,042 | 248,204 | 2,648,485 | 264,849 | 2,789,625 | 278,963 |  |
|  | 70 - 79 | 1,574,933 | 157,493 | 1,662,217 | 166,222 | 1,735,945 | 173,595 | 1,775,989 | 177,599 | 1,793,149 | 179,315 | 1,804,079 | 180,408 | 1,845,299 | 184,530 |  |
|  | Sum | 18,878,198 | 1,887,820 | 19,061,554 | 1,906,156 | 19,235,928 | 1,923,594 | 19,404,076 | 1,940,408 | 19,558,230 | 1,955,823 | 19,699,979 | 1,969,999 | 19,835,193 | 1,983,520 |  |
| Total | | 37,692,566 | 3,769,258 | 38,090,142 | 3,809,015 | 38,475,512 | 3,847,552 | 38,855,142 | 3,885,516 | 39,205,275 | 3,920,528 | 39,523,717 | 3,952,373 | 39,820,492 | 3,982,051 |  |
