# Supplementary material for: Impacts of smoking on alcoholic liver disease: a nationwide cohort study
Source: Front Public Health. 2024 Aug 7;12:1427131. doi: 10.3389/fpubh.2024.1427131 (PMC11335641; doi:10.3389/fpubh.2024.1427131)
Supplement: Supplementary file 3 [file Table_3.docx]

**Supplementary Table 3.** Prevalence of liver cirrhosis

|  | |  | | **Prevalence (%)** | | | | | | | | | | | | | |
| --- | --- | --- | --- | --- | --- | --- | --- | --- | --- | --- | --- | --- | --- | --- | --- | --- | --- |
| **Sex** | | **Age** | | **2011** | | **2012** | | **2013** | | **2014** | | **2015** | | **2016** | | **2017** | |
| **Social drinker** | | | | | | | | | | | | | | | | | |
| Male | | 20 – 29 | | 0.01 | | 0.01 | | 0.01 | | 0.01 | | 0.00 | | 0.01 | | 0.01 | |
|  |  | 30 – 39 | | 0.04 | | 0.02 | | 0.03 | | 0.04 | | 0.04 | | 0.03 | | 0.04 | |
|  |  | 40 – 49 | | 0.16 | | 0.15 | | 0.15 | | 0.16 | | 0.16 | | 0.15 | | 0.13 | |
|  |  | 50 – 59 | | 0.34 | | 0.35 | | 0.34 | | 0.41 | | 0.37 | | 0.35 | | 0.33 | |
|  |  | 60 – 69 | | 0.38 | | 0.40 | | 0.44 | | 0.36 | | 0.37 | | 0.45 | | 0.41 | |
|  |  | 70 – 79 | | 0.27 | | 0.33 | | 0.37 | | 0.38 | | 0.35 | | 0.45 | | 0.41 | |
|  |  | Sum | | 0.14 | | 0.15 | | 0.15 | | 0.17 | | 0.16 | | 0.17 | | 0.16 | |
| Female | | 20 – 29 | | 0.00 | | 0.00 | | 0.00 | | 0.00 | | 0.00 | | 0.01 | | 0.01 | |
|  |  | 30 – 39 | | 0.01 | | 0.01 | | 0.01 | | 0.01 | | 0.01 | | 0.01 | | 0.01 | |
|  |  | 40 – 49 | | 0.04 | | 0.05 | | 0.03 | | 0.04 | | 0.04 | | 0.04 | | 0.04 | |
|  |  | 50 – 59 | | 0.10 | | 0.10 | | 0.11 | | 0.12 | | 0.11 | | 0.12 | | 0.08 | |
|  |  | 60 – 69 | | 0.16 | | 0.15 | | 0.15 | | 0.16 | | 0.16 | | 0.18 | | 0.20 | |
|  |  | 70 – 79 | | 0.17 | | 0.13 | | 0.13 | | 0.15 | | 0.22 | | 0.14 | | 0.26 | |
|  |  | Sum | | 0.03 | | 0.04 | | 0.04 | | 0.04 | | 0.04 | | 0.05 | | 0.04 | |
| Total | | | | 0.09 | | 0.10 | | 0.10 | | 0.11 | | 0.11 | | 0.11 | | 0.11 | |
| **High risk drinker** | | | | | | | | | | | | | | | | |  |
| Male | 20 – 29 | | 0.01 | | 0.01 | | 0.01 | | 0.01 | | 0.00 | | 0.00 | | 0.01 | |  |
|  | 30 – 39 | | 0.02 | | 0.02 | | 0.02 | | 0.02 | | 0.03 | | 0.02 | | 0.02 | |  |
|  | 40 – 49 | | 0.15 | | 0.18 | | 0.16 | | 0.14 | | 0.14 | | 0.15 | | 0.15 | |  |
|  | 50 – 59 | | 0.40 | | 0.48 | | 0.44 | | 0.41 | | 0.41 | | 0.40 | | 0.37 | |  |
|  | 60 – 69 | | 0.72 | | 0.71 | | 0.70 | | 0.62 | | 0.68 | | 0.70 | | 0.65 | |  |
|  | 70 – 79 | | 0.92 | | 0.96 | | 0.80 | | 1.03 | | 0.64 | | 0.93 | | 0.92 | |  |
|  | Sum | | 0.20 | | 0.23 | | 0.22 | | 0.21 | | 0.21 | | 0.22 | | 0.22 | |  |
| Female | 20 – 29 | | 0.00 | | 0.00 | | 0.00 | | 0.01 | | 0.00 | | 0.00 | | 0.00 | |  |
|  | 30 – 39 | | 0.01 | | 0.02 | | 0.02 | | 0.05 | | 0.02 | | 0.01 | | 0.02 | |  |
|  | 40 – 49 | | 0.09 | | 0.12 | | 0.17 | | 0.13 | | 0.12 | | 0.17 | | 0.12 | |  |
|  | 50 – 59 | | 0.13 | | 0.27 | | 0.26 | | 0.32 | | 0.22 | | 0.26 | | 0.33 | |  |
|  | 60 – 69 | | 0.23 | | 0.40 | | 0.34 | | 0.40 | | 0.15 | | 0.34 | | 0.34 | |  |
|  | 70 – 79 | | 0.42 | | 0.20 | | 0.19 | | 0.17 | | 0.16 | | 0.32 | | 0.29 | |  |
|  | Sum | | 0.05 | | 0.08 | | 0.08 | | 0.09 | | 0.07 | | 0.09 | | 0.09 | |  |
| Total | | | 0.17 | | 0.20 | | 0.19 | | 0.19 | | 0.18 | | 0.20 | | 0.19 | |  |
| **High risk drinker with smoking** | | | | | | | | | | | | | | | | |  |
| Male | 20 – 29 | | 0.01 | | 0.01 | | 0.01 | | 0.00 | | 0.01 | | 0.00 | | 0.01 | |  |
|  | 30 – 39 | | 0.03 | | 0.02 | | 0.02 | | 0.02 | | 0.04 | | 0.03 | | 0.02 | |  |
|  | 40 – 49 | | 0.17 | | 0.22 | | 0.21 | | 0.17 | | 0.19 | | 0.20 | | 0.17 | |  |
|  | 50 – 59 | | 0.50 | | 0.64 | | 0.58 | | 0.58 | | 0.60 | | 0.55 | | 0.50 | |  |
|  | 60 – 69 | | 0.94 | | 0.89 | | 0.91 | | 0.80 | | 0.88 | | 0.91 | | 0.78 | |  |
|  | 70 – 79 | | 1.36 | | 1.22 | | 1.00 | | 1.26 | | 0.81 | | 1.39 | | 0.87 | |  |
|  | Sum | | 0.20 | | 0.24 | | 0.23 | | 0.23 | | 0.24 | | 0.25 | | 0.22 | |  |
| Female | 20 – 29 | | 0.01 | | 0.00 | | 0.00 | | 0.00 | | 0.00 | | 0.01 | | 0.01 | |  |
|  | 30 – 39 | | 0.07 | | 0.06 | | 0.07 | | 0.09 | | 0.08 | | 0.02 | | 0.08 | |  |
|  | 40 – 49 | | 0.19 | | 0.25 | | 0.40 | | 0.37 | | 0.32 | | 0.37 | | 0.27 | |  |
|  | 50 – 59 | | 0.26 | | 0.47 | | 0.27 | | 0.45 | | 0.26 | | 0.61 | | 0.65 | |  |
|  | 60 – 69 | | 0.54 | | 0.91 | | 0.63 | | 1.12 | | 0.18 | | 0.63 | | 0.52 | |  |
|  | 70 – 79 | | 1.61 | | 1.39 | | 0.00 | | 0.00 | | 0.00 | | 0.00 | | 0.00 | |  |
|  | Sum | | 0.11 | | 0.16 | | 0.16 | | 0.20 | | 0.14 | | 0.20 | | 0.19 | |  |
| Total | | | 0.20 | | 0.24 | | 0.23 | | 0.23 | | 0.23 | | 0.24 | | 0.22 | |  |
