# Supplementary material for: Impacts of smoking on alcoholic liver disease: a nationwide cohort study
Source: Front Public Health. 2024 Aug 7;12:1427131. doi: 10.3389/fpubh.2024.1427131 (PMC11335641; doi:10.3389/fpubh.2024.1427131)
Supplement: Supplementary file 4 [file Table_4.docx]

**Supplementary Table 4.** Prevalence of hepatocellular carcinoma

|  | |  | | **Prevalence (%)** | | | | | | | | | | | |
| --- | --- | --- | --- | --- | --- | --- | --- | --- | --- | --- | --- | --- | --- | --- | --- |
| **Sex** | | **Age** | | **2011** | **2012** | | **2013** | | **2014** | | **2015** | | **2016** | **2017** | |
| **Social drinker** | | | | | | | | | | | | | | | |
| Male | | 20 – 29 | | 0.00 | 0.00 | | 0.00 | | 0.00 | | 0.00 | | 0.00 | 0.00 | |
|  |  | 30 – 39 | | 0.00 | 0.01 | | 0.00 | | 0.01 | | 0.01 | | 0.01 | 0.00 | |
|  |  | 40 – 49 | | 0.04 | 0.04 | | 0.02 | | 0.03 | | 0.03 | | 0.04 | 0.04 | |
|  |  | 50 – 59 | | 0.10 | 0.12 | | 0.11 | | 0.11 | | 0.14 | | 0.12 | 0.11 | |
|  |  | 60 – 69 | | 0.14 | 0.16 | | 0.19 | | 0.20 | | 0.16 | | 0.19 | 0.23 | |
|  |  | 70 – 79 | | 0.12 | 0.25 | | 0.15 | | 0.26 | | 0.16 | | 0.23 | 0.25 | |
|  |  | Sum | | 0.04 | 0.05 | | 0.04 | | 0.06 | | 0.06 | | 0.06 | 0.07 | |
| Female | | 20 – 29 | | 0.00 | 0.00 | | 0.00 | | 0.00 | | 0.00 | | 0.00 | 0.00 | |
|  |  | 30 – 39 | | 0.00 | 0.00 | | 0.00 | | 0.00 | | 0.00 | | 0.00 | 0.00 | |
|  |  | 40 – 49 | | 0.01 | 0.01 | | 0.01 | | 0.01 | | 0.01 | | 0.01 | 0.01 | |
|  |  | 50 – 59 | | 0.02 | 0.01 | | 0.02 | | 0.03 | | 0.03 | | 0.03 | 0.03 | |
|  |  | 60 – 69 | | 0.04 | 0.02 | | 0.04 | | 0.05 | | 0.04 | | 0.03 | 0.03 | |
|  |  | 70 – 79 | | 0.09 | 0.06 | | 0.04 | | 0.06 | | 0.08 | | 0.04 | 0.09 | |
|  |  | Sum | | 0.01 | 0.01 | | 0.01 | | 0.01 | | 0.01 | | 0.01 | 0.01 | |
| Total | | | | 0.03 | 0.03 | | 0.03 | | 0.03 | | 0.03 | | 0.04 | 0.04 | |
| **High risk drinker** | | | | | | | | | | | | | | |  |
| Male | 20 – 29 | | 0.00 | | 0.00 | 0.00 | | 0.00 | | 0.00 | | 0.00 | | 0.00 |  |
|  | 30 – 39 | | 0.00 | | 0.01 | 0.00 | | 0.00 | | 0.00 | | 0.00 | | 0.01 |  |
|  | 40 – 49 | | 0.02 | | 0.02 | 0.03 | | 0.02 | | 0.03 | | 0.03 | | 0.02 |  |
|  | 50 – 59 | | 0.10 | | 0.10 | 0.11 | | 0.07 | | 0.09 | | 0.08 | | 0.08 |  |
|  | 60 – 69 | | 0.17 | | 0.16 | 0.20 | | 0.20 | | 0.19 | | 0.20 | | 0.19 |  |
|  | 70 – 79 | | 0.24 | | 0.21 | 0.33 | | 0.32 | | 0.28 | | 0.36 | | 0.30 |  |
|  | Sum | | 0.04 | | 0.05 | 0.06 | | 0.05 | | 0.05 | | 0.06 | | 0.05 |  |
| Female | 20 – 29 | | 0.00 | | 0.00 | 0.00 | | 0.00 | | 0.00 | | 0.00 | | 0.00 |  |
|  | 30 – 39 | | 0.00 | | 0.00 | 0.00 | | 0.00 | | 0.00 | | 0.00 | | 0.00 |  |
|  | 40 – 49 | | 0.00 | | 0.01 | 0.01 | | 0.00 | | 0.01 | | 0.00 | | 0.00 |  |
|  | 50 – 59 | | 0.04 | | 0.02 | 0.02 | | 0.05 | | 0.04 | | 0.06 | | 0.05 |  |
|  | 60 – 69 | | 0.19 | | 0.08 | 0.00 | | 0.07 | | 0.06 | | 0.05 | | 0.05 |  |
|  | 70 – 79 | | 0.21 | | 0.00 | 0.00 | | 0.00 | | 0.00 | | 0.16 | | 0.15 |  |
|  | Sum | | 0.01 | | 0.01 | 0.01 | | 0.01 | | 0.01 | | 0.01 | | 0.01 |  |
| Total | | | 0.04 | | 0.04 | 0.05 | | 0.04 | | 0.04 | | 0.05 | | 0.04 |  |
| **High risk drinker with smoking** | | | | | | | | | | | | | | |  |
| Male | 20 – 29 | | 0.00 | | 0.00 | 0.00 | | 0.00 | | 0.00 | | 0.00 | | 0.00 |  |
|  | 30 – 39 | | 0.00 | | 0.01 | 0.00 | | 0.00 | | 0.00 | | 0.01 | | 0.01 |  |
|  | 40 – 49 | | 0.02 | | 0.03 | 0.03 | | 0.03 | | 0.04 | | 0.03 | | 0.03 |  |
|  | 50 – 59 | | 0.12 | | 0.14 | 0.13 | | 0.08 | | 0.10 | | 0.11 | | 0.11 |  |
|  | 60 – 69 | | 0.22 | | 0.21 | 0.21 | | 0.23 | | 0.23 | | 0.24 | | 0.22 |  |
|  | 70 – 79 | | 0.50 | | 0.21 | 0.39 | | 0.19 | | 0.51 | | 0.53 | | 0.45 |  |
|  | Sum | | 0.04 | | 0.05 | 0.05 | | 0.04 | | 0.05 | | 0.05 | | 0.05 |  |
| Female | 20 – 29 | | 0.00 | | 0.00 | 0.00 | | 0.00 | | 0.00 | | 0.00 | | 0.00 |  |
|  | 30 – 39 | | 0.00 | | 0.00 | 0.00 | | 0.00 | | 0.00 | | 0.00 | | 0.00 |  |
|  | 40 – 49 | | 0.04 | | 0.04 | 0.00 | | 0.00 | | 0.02 | | 0.00 | | 0.00 |  |
|  | 50 – 59 | | 0.04 | | 0.04 | 0.04 | | 0.08 | | 0.08 | | 0.11 | | 0.07 |  |
|  | 60 – 69 | | 0.00 | | 0.00 | 0.00 | | 0.19 | | 0.00 | | 0.00 | | 0.00 |  |
|  | 70 – 79 | | 1.61 | | 0.00 | 0.00 | | 0.00 | | 0.00 | | 0.00 | | 0.00 |  |
|  | Sum | | 0.02 | | 0.01 | 0.00 | | 0.01 | | 0.01 | | 0.01 | | 0.01 |  |
| Total | | | 0.04 | | 0.05 | 0.05 | | 0.04 | | 0.05 | | 0.05 | | 0.05 |  |
