# Supplementary material for: Impacts of smoking on alcoholic liver disease: a nationwide cohort study
Source: Front Public Health. 2024 Aug 7;12:1427131. doi: 10.3389/fpubh.2024.1427131 (PMC11335641; doi:10.3389/fpubh.2024.1427131)
Supplement: Supplementary file 5 [file Table_5.docx]

**Supplementary Table 5.** 3-year mortality

|  |  | 2011  3-year follow-up  (2012-2014) | | | 2012  3-year follow-up  (2013-2015) | | | 2013  3-year follow-up  (2014-2016) | | | 2014  3-year follow-up  (2015-2017) | | | 2015  3-year follow-up  (2016-2018) | | | 2016  3-year follow-up  (2017-2019) | | | 2017  3-year follow-up  (2018-2020) | | |
| --- | --- | --- | --- | --- | --- | --- | --- | --- | --- | --- | --- | --- | --- | --- | --- | --- | --- | --- | --- | --- | --- | --- |
| Sex | Age | Cohort | Incidence  (n) | Incidence  rate (%) | Cohort | Incidence  (n) | Incidence  rate (%) | Cohort | Incidence  (n) | Incidence  rate (%) | Cohort | Incidence  (n) | Incidence  rate (%) | Cohort | Incidence  (n) | Incidence  rate (%) | Cohort | Incidence  (n) | Incidence  rate (%) | Cohort | Incidence  (n) | Incidence  rate (%) |
| **Social drinker** | | | | | | | | | | | | | | | | | | | | | | |
| Male | 20 – 29 | 72610 | 87 | 0.12 | 74378 | 74 | 0.10 | 76492 | 85 | 0.11 | 77237 | 79 | 0.10 | 75836 | 81 | 0.11 | 75221 | 72 | 0.10 | 74777 | 70 | 0.09 |
|  | 30 – 39 | 81979 | 117 | 0.14 | 84020 | 81 | 0.10 | 83540 | 95 | 0.11 | 84250 | 83 | 0.10 | 79588 | 89 | 0.11 | 76691 | 91 | 0.12 | 75951 | 69 | 0.09 |
|  | 40 – 49 | 71585 | 224 | 0.31 | 74268 | 234 | 0.32 | 74877 | 189 | 0.25 | 77993 | 182 | 0.23 | 76144 | 173 | 0.23 | 75135 | 178 | 0.24 | 75451 | 196 | 0.26 |
|  | 50 – 59 | 51378 | 349 | 0.68 | 56457 | 395 | 0.70 | 56843 | 385 | 0.68 | 61782 | 375 | 0.61 | 61630 | 334 | 0.54 | 61663 | 327 | 0.53 | 63744 | 340 | 0.53 |
|  | 60 – 69 | 24414 | 433 | 1.77 | 26051 | 446 | 1.71 | 26615 | 416 | 1.56 | 28979 | 449 | 1.55 | 31635 | 427 | 1.35 | 33669 | 426 | 1.27 | 36530 | 469 | 1.28 |
|  | Sum | 301966 | 1210 | 0.40 | 315174 | 1230 | 0.39 | 318367 | 1170 | 0.37 | 330241 | 1168 | 0.35 | 324833 | 1104 | 0.34 | 322379 | 1094 | 0.34 | 326453 | 1144 | 0.35 |
| Fe  male | 20 – 29 | 71020 | 29 | 0.04 | 71552 | 29 | 0.04 | 71674 | 29 | 0.04 | 74062 | 32 | 0.04 | 71950 | 30 | 0.04 | 71835 | 28 | 0.04 | 73380 | 29 | 0.04 |
|  | 30 – 39 | 80020 | 56 | 0.07 | 81336 | 48 | 0.06 | 81654 | 49 | 0.06 | 83570 | 47 | 0.06 | 82193 | 66 | 0.08 | 80331 | 37 | 0.05 | 81835 | 42 | 0.05 |
|  | 40 – 49 | 69289 | 87 | 0.13 | 70281 | 93 | 0.13 | 73174 | 76 | 0.10 | 77248 | 80 | 0.10 | 78023 | 88 | 0.11 | 77969 | 80 | 0.10 | 80804 | 69 | 0.09 |
|  | 50 – 59 | 33861 | 105 | 0.31 | 36777 | 95 | 0.26 | 39249 | 83 | 0.21 | 42843 | 92 | 0.21 | 45588 | 83 | 0.18 | 47031 | 98 | 0.21 | 50555 | 106 | 0.21 |
|  | 60 – 69 | 10476 | 65 | 0.62 | 10931 | 66 | 0.60 | 11346 | 62 | 0.55 | 12550 | 64 | 0.51 | 14074 | 60 | 0.43 | 15584 | 77 | 0.49 | 17465 | 89 | 0.51 |
|  | Sum | 264666 | 342 | 0.13 | 270877 | 331 | 0.12 | 277097 | 299 | 0.11 | 290273 | 315 | 0.11 | 291828 | 327 | 0.11 | 292750 | 320 | 0.11 | 304039 | 335 | 0.11 |
| Total | | c | 1552 | 0.27 | 586051 | 1561 | 0.27 | 595464 | 1469 | 0.25 | 620514 | 1483 | 0.24 | 616661 | 1431 | 0.23 | 615129 | 1414 | 0.23 | 630492 | 1479 | 0.23 |
| **High risk drinker** | | | | | | | | | | | | | | | | | | | | | | |
| Male | 20 – 29 | 88292 | 132 | 0.15 | 83677 | 135 | 0.16 | 80116 | 136 | 0.17 | 79156 | 117 | 0.15 | 80420 | 109 | 0.14 | 78307 | 93 | 0.12 | 78112 | 118 | 0.15 |
|  | 30 – 39 | 109604 | 208 | 0.19 | 105999 | 204 | 0.19 | 103386 | 193 | 0.19 | 100585 | 162 | 0.16 | 100064 | 155 | 0.15 | 96927 | 155 | 0.16 | 94946 | 175 | 0.18 |
|  | 40 – 49 | 131123 | 566 | 0.43 | 129136 | 612 | 0.47 | 128683 | 551 | 0.43 | 128185 | 513 | 0.40 | 129772 | 513 | 0.40 | 126875 | 506 | 0.40 | 124298 | 426 | 0.34 |
|  | 50 – 59 | 88020 | 1013 | 1.15 | 92556 | 962 | 1.04 | 97183 | 1036 | 1.07 | 100346 | 899 | 0.90 | 105648 | 931 | 0.88 | 107819 | 935 | 0.87 | 109781 | 928 | 0.85 |
|  | 60 – 69 | 32769 | 858 | 2.62 | 33471 | 833 | 2.49 | 34261 | 778 | 2.27 | 36497 | 813 | 2.23 | 39022 | 784 | 2.01 | 41696 | 809 | 1.94 | 44853 | 860 | 1.92 |
|  | Sum | 449808 | 2777 | 0.62 | 444839 | 2746 | 0.62 | 443629 | 2694 | 0.61 | 444769 | 2504 | 0.56 | 454926 | 2492 | 0.55 | 451624 | 2498 | 0.55 | 451990 | 2507 | 0.55 |
| Fe  male | 20 – 29 | 37311 | 32 | 0.09 | 37242 | 29 | 0.08 | 37583 | 25 | 0.07 | 39151 | 18 | 0.05 | 42458 | 31 | 0.07 | 42281 | 34 | 0.08 | 44128 | 37 | 0.08 |
|  | 30 – 39 | 27230 | 58 | 0.21 | 25213 | 52 | 0.21 | 28646 | 35 | 0.12 | 26550 | 41 | 0.15 | 29491 | 42 | 0.14 | 29812 | 36 | 0.12 | 30954 | 50 | 0.16 |
|  | 40 – 49 | 21206 | 61 | 0.29 | 21562 | 68 | 0.32 | 22821 | 74 | 0.32 | 23901 | 63 | 0.26 | 25806 | 64 | 0.25 | 26597 | 76 | 0.29 | 27396 | 90 | 0.33 |
|  | 50 – 59 | 11350 | 62 | 0.55 | 12023 | 57 | 0.47 | 12745 | 73 | 0.57 | 13011 | 65 | 0.50 | 14229 | 70 | 0.49 | 14527 | 54 | 0.37 | 15059 | 69 | 0.46 |
|  | 60 – 69 | 2133 | 22 | 1.03 | 2509 | 36 | 1.43 | 2621 | 20 | 0.76 | 3023 | 32 | 1.06 | 3401 | 32 | 0.94 | 3772 | 35 | 0.93 | 4351 | 40 | 0.92 |
|  | Sum | 99230 | 235 | 0.24 | 98549 | 242 | 0.25 | 104416 | 227 | 0.22 | 105636 | 219 | 0.21 | 115385 | 239 | 0.21 | 116989 | 235 | 0.20 | 121888 | 286 | 0.23 |
| Total | | 549038 | 3012 | 0.55 | 543388 | 2988 | 0.55 | 548045 | 2921 | 0.53 | 550405 | 2723 | 0.49 | 570311 | 2731 | 0.48 | 568613 | 2733 | 0.48 | 573878 | 2793 | 0.49 |
| **High risk drinker with smoking** | | | | | | | | | | | | | | | | | | | | | | |
| Male | 20 – 29 | 59891 | 111 | 0.19 | 56018 | 108 | 0.19 | 52693 | 103 | 0.20 | 50377 | 96 | 0.19 | 49594 | 77 | 0.16 | 48549 | 70 | 0.14 | 47626 | 87 | 0.18 |
|  | 30 – 39 | 73835 | 157 | 0.21 | 71023 | 164 | 0.23 | 69141 | 141 | 0.20 | 65349 | 130 | 0.20 | 61396 | 115 | 0.19 | 60023 | 122 | 0.20 | 57161 | 117 | 0.20 |
|  | 40 – 49 | 77198 | 423 | 0.55 | 76010 | 463 | 0.61 | 76264 | 417 | 0.55 | 75879 | 409 | 0.54 | 73277 | 364 | 0.50 | 74310 | 402 | 0.54 | 72054 | 317 | 0.44 |
|  | 50 – 59 | 43672 | 680 | 1.56 | 45323 | 670 | 1.48 | 48095 | 735 | 1.53 | 48386 | 627 | 1.30 | 47681 | 637 | 1.34 | 50992 | 622 | 1.22 | 51874 | 626 | 1.21 |
|  | 60 – 69 | 13042 | 467 | 3.58 | 13432 | 467 | 3.48 | 13578 | 421 | 3.10 | 14234 | 459 | 3.22 | 13601 | 406 | 2.99 | 15361 | 407 | 2.65 | 16601 | 458 | 2.76 |
|  | Sum | 267638 | 1838 | 0.69 | 261806 | 1872 | 0.72 | 259771 | 1817 | 0.70 | 254225 | 1721 | 0.68 | 245549 | 1599 | 0.65 | 249235 | 1623 | 0.65 | 245316 | 1605 | 0.65 |
| Fe  male | 20 – 29 | 7223 | 8 | 0.11 | 7180 | 10 | 0.14 | 7275 | 10 | 0.14 | 7048 | 8 | 0.11 | 7363 | 12 | 0.16 | 7607 | 14 | 0.18 | 8142 | 9 | 0.11 |
|  | 30 – 39 | 5808 | 18 | 0.31 | 5267 | 17 | 0.32 | 5976 | 12 | 0.20 | 5279 | 18 | 0.34 | 5332 | 13 | 0.24 | 5793 | 12 | 0.21 | 5943 | 17 | 0.29 |
|  | 40 – 49 | 4639 | 33 | 0.71 | 4774 | 33 | 0.69 | 4981 | 33 | 0.66 | 5166 | 23 | 0.45 | 5338 | 29 | 0.54 | 5900 | 27 | 0.46 | 5986 | 38 | 0.63 |
|  | 50 – 59 | 2344 | 22 | 0.94 | 2539 | 29 | 1.14 | 2637 | 18 | 0.68 | 2654 | 31 | 1.17 | 2648 | 24 | 0.91 | 2793 | 22 | 0.79 | 3058 | 27 | 0.88 |
|  | 60 – 69 | 367 | 8 | 2.18 | 441 | 12 | 2.72 | 477 | 7 | 1.47 | 538 | 10 | 1.86 | 546 | 10 | 1.83 | 634 | 13 | 2.05 | 766 | 16 | 2.09 |
|  | Sum | 20381 | 89 | 0.44 | 20201 | 101 | 0.50 | 21346 | 80 | 0.37 | 20685 | 90 | 0.44 | 21227 | 88 | 0.41 | 22727 | 88 | 0.39 | 23895 | 107 | 0.45 |
| Total | | 288019 | 1927 | 0.67 | 282007 | 1973 | 0.70 | 281117 | 1897 | 0.67 | 274910 | 1811 | 0.66 | 266776 | 1687 | 0.63 | 271962 | 1711 | 0.63 | 269211 | 1712 | 0.64 |
