# Supplementary figures and images for: Impacts of smoking on alcoholic liver disease: a nationwide cohort study
Source: Front Public Health. 2024 Aug 7;12:1427131. doi: 10.3389/fpubh.2024.1427131 (PMC11335641; doi:10.3389/fpubh.2024.1427131)

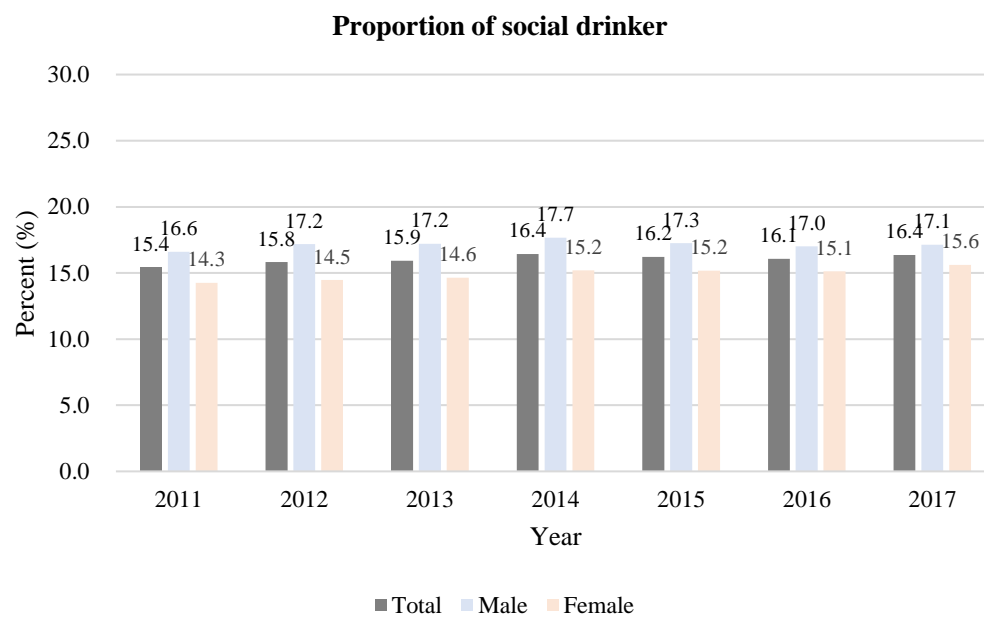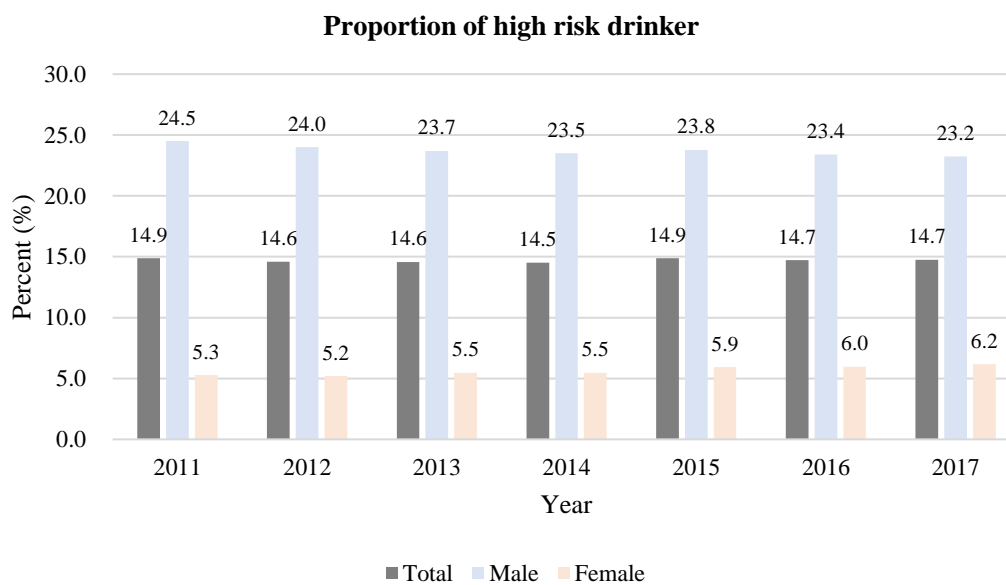

**Supplementary Figure 1. Alcohol consumption rate**

Supplement: Supplementary file 6 [file Image_1.pdf]

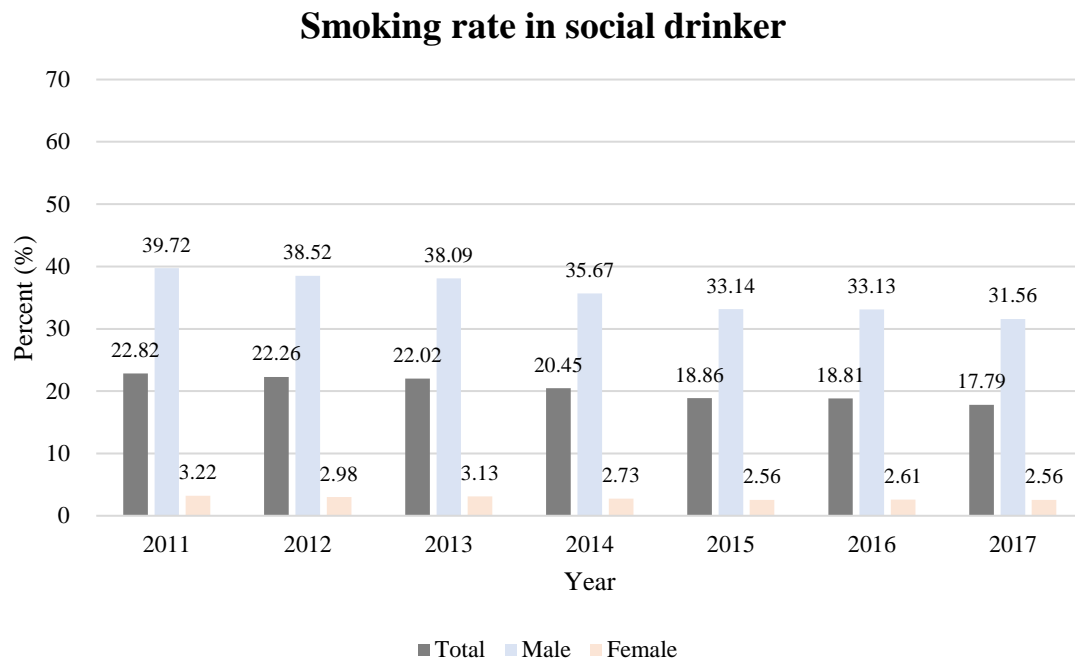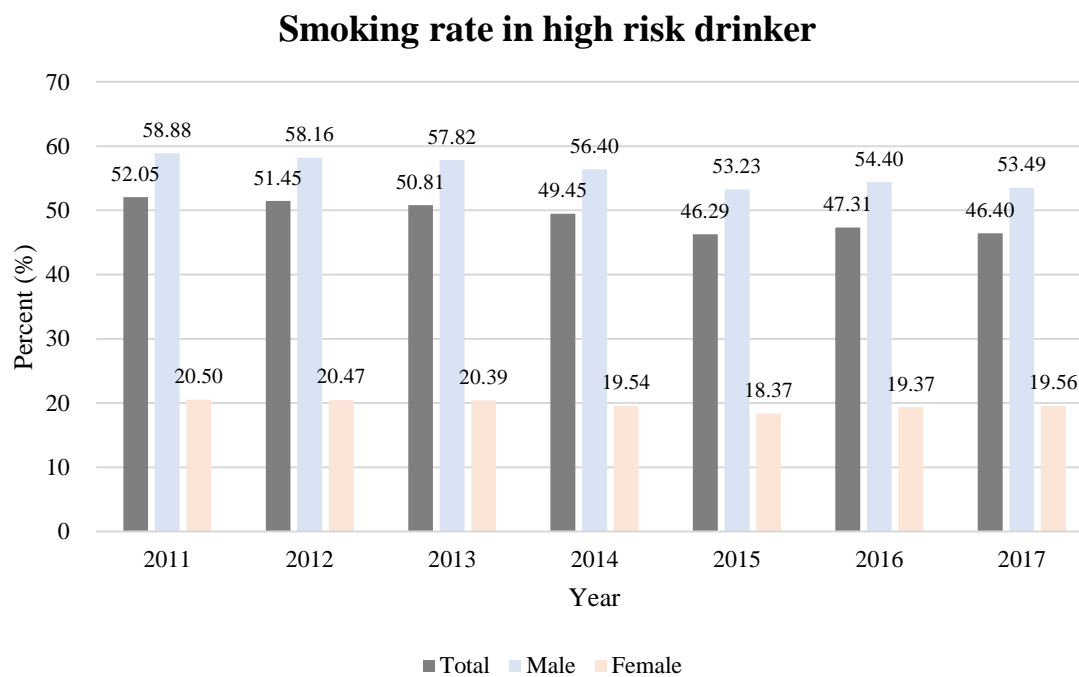

**Supplementary Figure 2.** Smoking rate in social drinker and high risk drinker

Supplement: Supplementary file 7 [file Image_2.pdf]
